# Bilateral Absence of the Superior Vena Cava

**DOI:** 10.1155/2012/461040

**Published:** 2012-06-20

**Authors:** Kaisa Ylänen, Tuija Poutanen, Päivi Savikurki-Heikkilä, Jukka Uotila, Matti Korppi, Anneli Eerola

**Affiliations:** ^1^Pediatric Research Centre, Tampere University Hospital, Finland; ^2^Department of Pediatrics, Tampere University Hospital, 33521 Tampere, Finland; ^3^Department of Radiology, Tampere University Hospital, Finland; ^4^Department of Obstetrics and Gynecology, Tampere University Hospital, Finland

## Abstract

Bilateral absence of the superior vena cava (SVC) is a very rarely detected, mainly asymptomatic congenital vascular anomaly. Though usually innocent, this anomaly may complicate cardiothoracic surgery and certain procedures like central venous catheter insertion. This SVC anomaly is poorly known, and we assume that its incidence in the general population may be higher than detected. In this paper, we summarize current knowledge on this anomaly and its clinical implications. In addition, we present a neonatal case with bilateral absence of the SVC associated with a fetal cystic hygroma. *Conclusion*. Totally absent SVC can cause unexpected problems during cardiothoracic surgery. Suspicion of SVC absence should arise in basic echocardiography. Our paper suggests that, like other congenital anomalies, bilateral absent SVC may be associated with a fetal cyctic hygroma.

## 1. Introduction

Congenital anomalies of the superior vena cava (SVC) are not uncommon and often coexist with other congenital cardiac anomalies. The most common of the SVC anomalies is bilateral SVC, which means that both right and left superior caval veins are present. Both bilateral SVC and isolated left SVC can occur in the otherwise normal heart or be associated with congenital heart disease [1]. If the left SVC drains into the right atrium, venous blood return into the heart is normal. For this reason, these SVC anomalies are asymptomatic and found incidentally. These anomalies may, however, complicate catheterization, central venous access, transvenous pacemaker implantation, and cardiac surgery. Bilateral SVC absence is a unique form of the SVC anomalies.

## 2. Case Report

**Figure 1:**
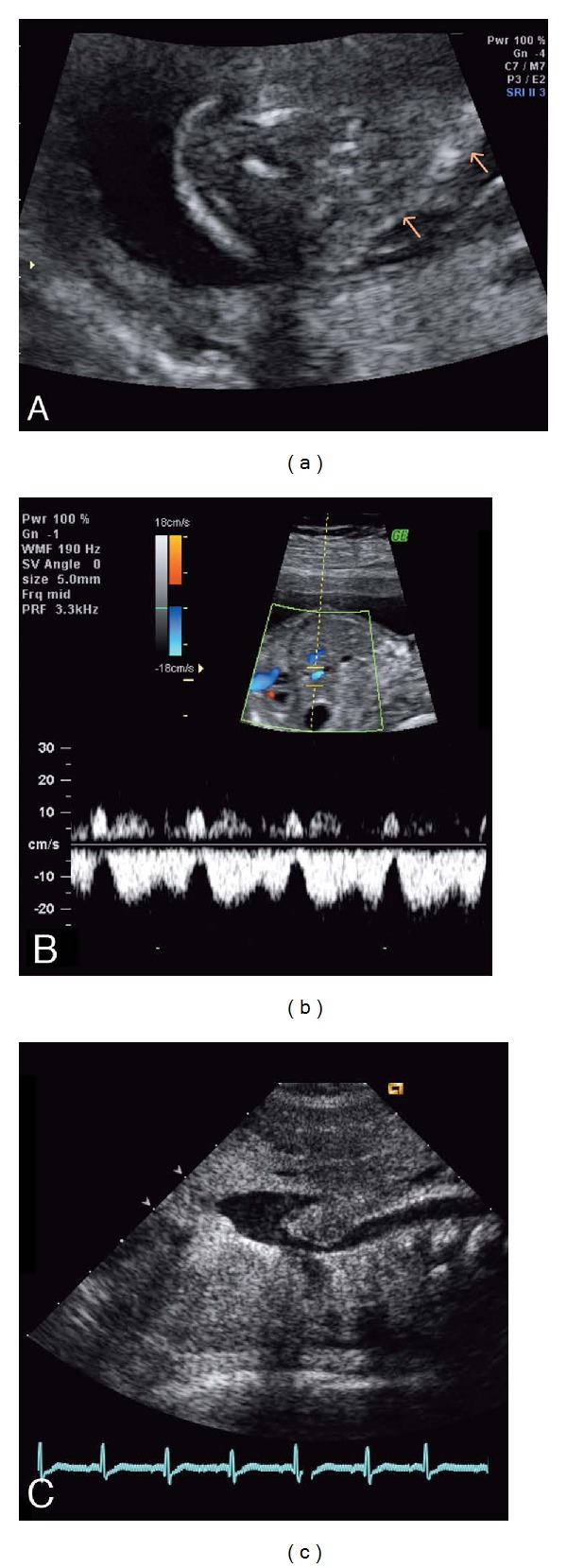
Ultrasound images prenatally and postnatally. (a) Fetal scan at the 12th week of pregnancy demonstrating moderate nuchal edema and cystic hygroma. (b) Pathological flow of the venous duct at the 20th week. During atrial contractions, a reverse flow is noticeable. (c) Two-dimensional echocardiogram of a newborn with absent SVC. The azygos and the hemiazygos veins have fused, and the venous duct formed is draining into the IVC.

The first-trimester ultrasound for a pregnant 28-year-old woman revealed cystic fetal hygroma, which resolved spontaneously by the 20th gestational week (Figure 1(a)). Placental villous sampling revealed a normal male karyotype (46, XY), and tests for congenital viral infection were all negative. The fetal heart seemed normal, but the pathological flow of the venous duct was noticeable at the 20th week of gestation, with reversed a-wave during atrial contraction (Figure 1(b)). Later in gestation this finding also normalized. The child was born at 39 + 4 weeks of gestation, weighing 4385 g. There were no dysmorphic features; only the head circumference was +3.4 standard deviation compared with national references. He needed supplemental oxygen for a few hours after delivery and thereafter was in good condition. No cardiac murmur was audible. The electrocardiogram and the chest X-ray were normal.

**Figure 2:**
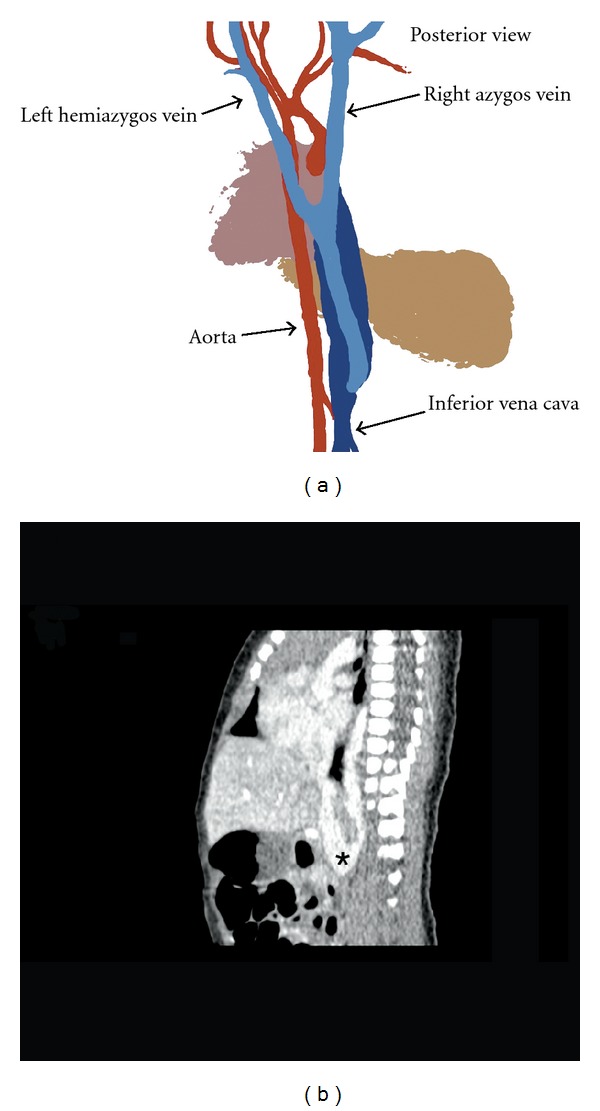
(a) Schematic drawing of azygos vein and hemiazygos vein fusion in the thoracic cavity. The venous duct formed is draining into the IVC. (b) Sagittal CT scan showing venous duct draining into the IVC (asterix).

Because of the hygroma detected by prenatal ultrasound, a transthoracic echocardiography was performed at the age of five days. It revealed normal structure and function of the heart except that neither left nor right superior caval veins existed. Instead, a venous structure was draining from the upper trunk behind the heart and joining the exceptionally broad inferior vena cava (IVC) that was connected to the right atrium (Figure 1(c)). The computerized tomography (CT) of the chest and abdomen ascertained five days later the anatomy; there was no SVC, and the blood from the head and the upper limbs drained into the right atrium through a venous plexus connected to the IVC (Figure 2). CT scanning was chosen as the imagining modality because of a relatively short imaging time to be performed under light sedation without need for intubation anesthesia. 

**Figure 3:**
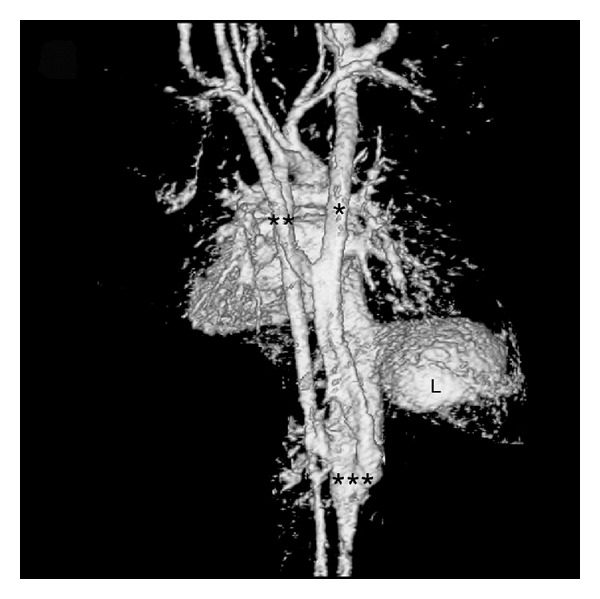
3D MRA construction of bilateral SVC absence from a posterior view showing the aorta and venous structures around the diaphragm. *Right azygos vein. **Left hemiazygos vein. ***Fused azygos and hemiazygos veins joining the IVC. Liver (L).

The boy had suffered from continuous wheezing since the age of one month, and symptoms worsened during respiratory infections. Because respiratory symptoms continued despite inhaled bronchodilators and corticosteroids, trachea compression was suspected, and at the age of eight months, the magnetic resonance angiography (MRA) of the thorax and abdomen was performed. The MRA confirmed the total absence of a SVC. Blood from the right arm and the right side of the head drained into the azygos vein, and blood from the left arm and the left side of the head drained into the hemiazygos vein. The azygos and the hemiazygos veins were fused below the level of the trachea bifurcation, and this vein formed a loop beneath the diaphragm joining the prominent IVC at the level of the kidneys, but both renal veins drained into the IVC above the conjunction (Figure 3). All other vessels were normal. No structures were compressing the trachea. Ultrasound of the upper abdomen was performed at the age of one year, showing no signs of portal hypertension.

We performed a number of genetic tests to exclude genetic syndromes connecting total absence of the SVC with large head circumference and the fetal cystic hygroma. The child had a normal male karyotype (46, the XY), and tests for CATCH 22 syndrome, Sotos syndrome, and Prader-Willi syndrome were all negative.

## 3. Discussion and Review

The venous system starts developing during the fourth week of embryonic life. The symmetrical cardinal veins and their tributaries form the embryonic superior and inferior caval system. The left and right anterior (superior caval system) cardinal veins drain the cephalic part of the embryo, and the left and right posterior (inferior caval system) cardinal veins drain the rest of the embryo. These cardinal veins join to form the right and left common cardinal veins before entering the right and left horns of the sinus venosus and further into the embryological heart. During the development of the venous system branching, regression, and anastomosing occur, resulting in the unilateral right-sided systemic venous return in most individuals. The SVC is formed by the right common cardinal vein and the proximal portion of the right anterior cardinal vein. Venous blood from the head, the upper limbs, and the upper half of the body drains into the right atrium via the right SVC, and the venous return from the left side of the upper trunk is connected to the SVC via the left brachiocephalic vein. An isolated left SVC arises from the left anterior cardinal vein with complete regression of the right. Bilateral SVC is characterized by the persistence of the right and left anterior cardinal veins. Disturbed development of the right common cardinal vein and the right anterior cardinal vein leads to the bilateral absence of SVC. 

Bilateral SVC occurs in 3 to 11% of patients with congenital heart disease compared with 0.3% in the general population [12, 13]. In situs abnormalities, however, the occurrence rates have been as high as 50 to 70% [12, 14]. Absence of the right SVC in visceroatrial situs solitus has been detectable postmortem in 0.09% of patients with congenital heart disease, and among the 121 cases reviewed with an absent right SVC, 54% had an otherwise normal heart structure [1]. SVC anomalies are usually asymptomatic, but they may complicate procedures like cardiac catheterization, systemic venous cannulation for extracorporeal membrane oxygenation, and transvenous pacemaker implantation. Cardiac surgery for cardiopulmonary bypass, partial or total cavopulmonary anastomoses, and orthotropic heart transplantation, for example, require exact knowledge of systemic venous anatomy. 

## 4. Clinical Presentation

We describe a very rare SVC anomaly with a totally absent SVC. To our knowledge, only ten previously cases are published that show the total absence or severe hypoplasia of the SVC (Table 1) [2–11]. The first two were adults, diagnosed by venography during an attempt to implant a pacemaker [2, 3]. One adult case was diagnosed because of complicated vascular access during an electrophysiological evaluation for ablation purpose [11]. Two other cases in adults were found incidentally with symptoms not related to an SVC anomaly [4, 8]. In addition, absence of the SVC has been associated with tetralogy of Fallot in one child [6] and with an atrial septal defect in another child [9]. Two newborns with absent SVC suffered from chylothorax, one detected prenatally and one postnatally [7, 9]. The cause of chylothorax in these cases was probably the poor venous return from the upper trunk, leading to high venous pressure. A male preponderance exists, both in total absence of the SVC (male: female ratio 6 : 3) and in absent right SVC [1]. Lytrivi et al. described an infant with a large head, cyanosis, and congenital atresia of the SVC with anomalous drainage of the upper body venous return to the left atrium through stenotic anastomoses between systemic and pulmonary veins [15]. The large head circumference may have been due to cyanosis-related increased hematopoiesis in the skull bone marrow, because after surgical correction of the venous return, the cyanosis resolved and head circumference normalized. Another mechanism causing increased head circumference with absent SVC is soft tissue edema due to SVC obstruction syndrome. The reason for our patient's large head remains unclear, because he had no right to left shunting nor SVC obstruction syndrome.

In patients with bilateral absent SVC, systemic venous blood return varies. Three cases had a left brachiocephalic vein or a transverse communicating vein between the left internal jugular vein and the azygos vein forming an anastomosis between the left and right on the cranial side of the aortic arch [2, 4, 7]. In the other four cases, as in the present case, an anastomosis between the left and right was only in the azygos system, with no upper communicating vein caudal to the aortic arch [5, 6, 8, 10]. Four different types of connections have been described from the azygos system into the right atrium: the azygos veins drain directly into the IVC, as in the present case [3, 5, 6, 10], and they drain into the left renal vein [8, 9], into the right atrium as in case of the absence of the hepatic segment of the IVC with azygos continuation [11], into the right atrium via a complex subdiaphragmatic plexus of veins without a clear, demonstrable communication with the IVC [2].

Fetal cystic hygroma is a congenital malformation in which fluid-filled spaces develop in the region of the fetal neck. Septated cystic hygroma diagnosed in early pregnancy is usually associated with chromosomal abnormalities and other major malformations like congenital heart disease. The risk is 5-fold for chromosomal abnormalities, 12-fold for cardiac malformation, and 6-fold for perinatal death, when compared with simple increased nuchal translucency [16]. In the present case, the first-trimester hygroma was the only reason for the cardiac examination of this neonate. Although his head circumference exceeded the normal limits for a newborn, no other extrinsic abnormalities were evident. Neither did the physical examination by the pediatric geneticist or genetic tests reveal suspicion of any genetic syndrome. This association between first-trimester cystic hygroma and total absence of the SVC has not been reported previously and remains unclear, but it is probable that anomalous venous drainage during early fetal life disturbed the fluid return and resulted in nuchal edema and cystic hygroma.

## 5. Diagnosis and Differential Diagnosis

 Patients with SVC obstruction syndrome usually present with facial, neck and arm swelling, superficial vein distension of the upper trunk, and dyspnoea at rest. SVC obstruction syndrome is usually caused by extrinsic compression of the SVC due to tumor or lymphadenopathy or complications following heart surgery. SVC obstruction may also be associated with central venous catheter insertion and thrombosis. Rarely, congenital SVC anomalies may cause SVC obstruction syndrome, and then symptoms usually manifest early in infancy in the case of underdevelopment of the collateral circulation [7].

Suspicion of SVC absence may arise in echocardiography, as happened in the present case. Echocardiography is always indicated if a newborn has signs or symptoms presumptive for congenital heart defect or if there is prenatal suspicion of a congenital heart defect. In addition, prenatally detected fetal swelling of unknown etiology, hydrops, hygroma, or a prominent nuchal translucency, even if resolved before delivery, is an indication for postnatal echocardiography. During routine echocardiography, it is important to positively identify all the normal structures of the heart from venous inflow to arterial outflow, being aware of potential congenital malformations, and if any of the normal structures cannot be visualized, to react accordingly. Echocardiography and abdominal ultrasound are nowadays the first-line diagnostic tools; they are noninvasive with no exposure to radiation and show high degree of accuracy. Because this SVC anomaly consists of venous structures around the diaphragm, it is often impossible to achieve a clear understanding of the anatomy of the veins with an ultrasound examination only. Conventional angiography is not the first line additional imagining method nowadays, because of its invasiveness and use of ionizing radiation. CT and MRI scanning are equally good in revealing anomalies of the venous structures. Benefits of MRI are its lack of radiation and the possibility to obtain good-quality three-dimensional pictures at the venous stage. The advantage of CT examinations is the short duration, making intubation unnecessary even in infants. Differential diagnosis includes acquired SVC abnormalities such as extrinsic compression by tumors, enlarged lymph nodes, vascular abnormalities, invasion of the SVC by tumors, or occlusion of the SVC by a catheter-associated thrombus [17].

## 6. Therapy and Prognosis

To our knowledge, no studies reported on any intervention or surgical correction of this condition without right to left shunting. This may be due to the fact that most patients are asymptomatic. Assessment of venous pressure in the systemic veins has been done in only one case [11]. Because constantly high pressure in the IVC due to increased blood volume may predispose to portal hypertension, we recommend for these patients regular and long-term cardiological followup and abdominal ultrasound examination. An 82-year-old woman with bilateral absence of the SVC had esophageal and gastric varices [2], but their association with her SVC anomaly remained unclear. An absent right SVC in visceroatrial situs solitus may be associated with abnormalities in the sinoatrial and atrioventricular nodes and offers an increased arrhythmia risk [1], necessitating electrocardiographic followup. Early diagnoses in newborns facilitate collection of long-term follow-up data and lead to increased knowledge of the natural course of this anomaly.

## 7. Conclusion

 A totally absent SVC is a very rare anomaly. Because it is usually asymptomatic, the incidence in the general population may be higher than detected. Despite the benign nature of this SVC anomaly, knowledge of the systemic venous anatomy has to be accurate prior to cardiac surgery. In case of totally absent SVC, some palliative procedures for univentricular heart defect, for example, bidirectional Glenn or total cavopulmonary connection, are difficult or even impossible to perform. Clamping of the IVC during hepatectomy for liver transplantation has devastating consequences due to obstructed venous return, when the bilateral absence of SVC has not been detected before operation. The possibility of this rare SVC anomaly should also be kept in mind when unexpected difficulties arise in systemic venous cannulation. Routinely used approach in pacemaker implantation cannot be used when SVC does not exist. Prenatal findings of congenital hydrothorax as well as septated cystic hygroma, even if spontaneously resolved during fetal life, are indications for cardiac evaluation during the neonatal period. The neonatologist must be aware of and well informed about fetal findings. If doubts emerge regarding systemic venous drainage, as a substitute the previous gold standard, invasive angiography, CT, or MRI is a necessary supplement to echocardiography.

## Figures and Tables

**Table 1:** Summary of the case reports published on bilateral absence of the superior vena cava (SVC).

Author	Year	Age at diagnosis; gender	Symptoms	Diagnostic examination	Intracardiac malformations	Other
Hussain et al. [2]	1981	82 years; female	Diagnosed during transvenous pacemaker placement (complete atrioventricular block)	Venography	No	Possibility of communication with intrahepatic circulation and formation of esophageal and gastric varices was also evident
Del Ojo et al. [3]	1999	81 years; male	Diagnosed during transvenous pacemaker placement (complete atrioventricular block)	Venography	No	Superficial varicose vein circulation at abdominal thoracic level
Saunders et al. [4]	2001	25 years; female	Mild dyspnea on exertion	MRI Venography Exploratory median sternotomy	No	
Minniti et al. [5]	2002	NA	NA	CT Venography	NA	
Krasemann et al. [6]	2003	3 months; male	Clinical signs of TOF	Catheterization Venography Operation	TOF	IVC also anomalous with an inferior part of the IVC on left side of the spine
Lee et al. [7]	2005	One week; female	Facial and upper trunk edema Respiratory distress Chylothorax	Echocardiography CT Venography Right atrium cardiography	No	Severe SVC dysplasia with multiple segments of stenosis at the SCV Symptoms persisting more than 6 months despite chylothorax improvement
Akai et al. [8]	2006	28 years; male	No	CT MRI	No	
Römer et al. [9]	2006	Prenatally at gestation week 24 + 5; male	Respiratory distress Chylothorax	Echocardiography MRI angiography	Type II and sinus venosus ASD	Symptoms resolved after pleural puncture
Ou et al. [10]	2007	14 months; male	Cardiac murmur	Echocardiography CT	No	
Quraishi et al. [11]	2010	59 years; male	Diagnosed during electrophysiological evaluation (WPW syndrome)	Venography CT angiography	No	Absence of hepatic segment of the IVC with azygos continuation

ASD: atrial septal defect; IVC: inferior vena cava; NA: not available; TOF: tetralogy of Fallot; WPW: Wolff-Parkinson-White.

## Abbreviations

CT: Computerized tomography
IVC: Inferior vena cava
MRA: Magnetic resonance angiography
SVC: Superior vena cava.
